# Real‐World Maintenance of Remission in Atopic Dermatitis Patients With Upadacitinib: A Multicenter Retrospective Study in Japan (ROADMAP Study)

**DOI:** 10.1111/1346-8138.70299

**Published:** 2026-06-04

**Authors:** Yuichiro Tsunemi, Atsuyuki Igarashi, Takeshi Ishido, Hidehisa Saeki

**Affiliations:** ^1^ Department of Dermatology Saitama Medical University Saitama Japan; ^2^ Igarashi Dermatology Higashigotanda Tokyo Japan; ^3^ Medical Affairs AbbVie GK Tokyo Japan; ^4^ Department of Dermatology Nippon Medical School Tokyo Japan

**Keywords:** atopic dermatitis, Janus kinase inhibitor, recurrence, treatment outcome, upadacitinib

## Abstract

Upadacitinib (UPA) is a selective Janus kinase 1 inhibitor that improves moderate‐to‐severe atopic dermatitis, but real‐world strategies to maintain remission after response remain unclear. We conducted a multicenter retrospective study in Japan of 219 patients aged ≥ 12 years who achieved protocol‐defined remission, defined as Investigator's Global Assessment (IGA) 0/1 at two visits ≥ 4 weeks apart during UPA treatment. Patients were classified as on‐label (continuous daily 15 or 30 mg), spacing/reduction (extension of the dosing interval and/or dose‐reduction after remission), or discontinuation (stopping UPA after remission). The primary endpoint was maintenance of remission over 48 weeks; secondary endpoints were time to relapse, outcomes after treatment modification, remission at 72 weeks after ≥ 24 weeks of sustained remission, and safety. Because the analysis was restricted to responders who achieved remission, generalizability to all UPA‐treated patients is limited. Seventy‐five percent of patients achieved IGA 0/1 within about 4 months of starting UPA. Over the subsequent 48 weeks, 127/200 (63.5%, 95% CI 56.6–69.9) maintained IGA 0/1. When stratified by post‐remission dosing strategy (*n* = 193; excluding 7 who switched to other systemic therapy), remission at 48 weeks was maintained in 93/145 (64.1%, 95% CI 55.8–71.9) in the on‐label group, 24/32 (75.0%, 95% CI 56.6–88.5) in the spacing/reduction group, and 6/16 (37.5%, 95% CI 15.2–64.6) in the discontinuation group. Among patients with ≥ 24 weeks of sustained remission, week‐72 IGA 0/1 maintenance rates were 70.7% (95% CI 60.7–79.1; *n* = 107), 100% (95% CI 63.1–100.0; *n* = 8), and 60.0% (95% CI 20.0–90.0; *n* = 5) in the on‐label, spacing/reduction, and discontinuation groups, respectively; estimates for spacing/reduction and discontinuation were imprecise due to small sample sizes. Longer on‐label treatment before tapering or discontinuation tended to be associated with a lower relapse risk. UPA showed a safety profile consistent with previous studies. Acne, herpes simplex, herpes zoster, and folliculitis were the most frequent adverse events, and most were mild and manageable, although one herpes zoster case required temporary interruption. In this responder‐enriched real‐world Japanese cohort, UPA induced remission in most patients within 4 months and allowed approximately two‐thirds to maintain remission over 48 weeks. Observed outcomes suggest that maintaining on‐label dosing and cautious spacing/reduction after sustained remission may help preserve disease control, whereas discontinuation may be associated with a higher risk of relapse; however, comparisons between dosing strategies are descriptive and hypothesis‐generating. Prospective studies are needed to confirm these findings.

## 1 Introduction

Atopic dermatitis (AD) is a chronic, relapsing inflammatory skin disease characterized by intense pruritus, eczematous lesions, and fluctuating severity over time. The Japanese clinical practice guidelines for AD in 2024 emphasize three primary management goals: achieving minimal or no symptoms, maintaining long‐term disease control, and minimizing pharmacologic intervention [1]. While topical corticosteroids (TCS) and calcineurin inhibitors remain the mainstay of treatment, newer topical agents such as delgocitinib, difamilast, and tapinarof are now also approved in Japan. However, patients with moderate‐to‐severe AD often require systemic therapy when the response to topical treatments is inadequate.

Conventional systemic agents, including oral corticosteroids and cyclosporine, have been approved for decades but their continuous use remains limited by toxicity, tolerability, and the risk of worsening after discontinuation. More recently, targeted systemic therapies, such as biologics and oral small‐molecule Janus kinase (JAK) inhibitors, have expanded treatment options and enabled long‐term disease control in many patients [2, 3]. Upadacitinib (UPA), a once‐daily, orally administered selective JAK1 inhibitor, has demonstrated rapid symptomatic relief and consistent improvements in disease‐severity scores such as the Eczema Area and Severity Index (EASI) and Investigator's Global Assessment (IGA), as well as quality‐of‐life (QoL) measures and patient‐reported outcomes across randomized trials, including the Phase 3 Measure UP program [4, 5, 6, 7, 8].

In Japan, guidance from the Ministry of Health, Labour and Welfare (MHLW) for targeted systemic therapies for AD (including UPA) indicates that, once sustained remission is achieved–particularly with concomitant topical therapy–clinicians may consider continued labeled dosing, cautious dose spacing/reduction (extension of the dosing interval and/or dose‐reduction after remission), or discontinuation after approximately 24 weeks [9]. However, these consensus‐based documents do not explicitly define “sustained remission” and provide limited operational criteria for related terms such as “relapse” or “dose tapering”; implementation therefore varies across institutions, and clinicians must balance disease control with treatment burden on a case‐by‐case basis.

To generate evidence and inform the use of UPA in patients with AD in real‐world Japanese clinical practice, we conducted a multicenter, retrospective observational study to examine UPA treatment patterns and long‐term disease control in patients who initially achieved prespecified clinical response. By focusing on outcomes over a follow‐up period of at least 48 weeks, this study aims to provide practical evidence—a “ROADMAP”—to guide decision‐making on the long‐term use of UPA among treatment responders, specifically regarding the maintenance of clinical response by dosing strategy (continuous dosing, dose tapering/spacing, or discontinuation).

## 2 Methods

### 2.1 Study Subjects

Patients who visited participating medical institutions between August 2021 and March 2024 and fulfilled all the following criteria were enrolled: (1) a diagnosis of AD and treatment with UPA; (2) age ≥ 12 years; (3) achievement of protocol‐defined remission; and (4) availability of verifiable medical records and at least 48 weeks of documented follow‐up from achievement of remission.

An opt‐out process was implemented at all sites; patients who opted out were excluded, and their records were not analyzed.

### 2.2 Study Design

**FIGURE 1:**
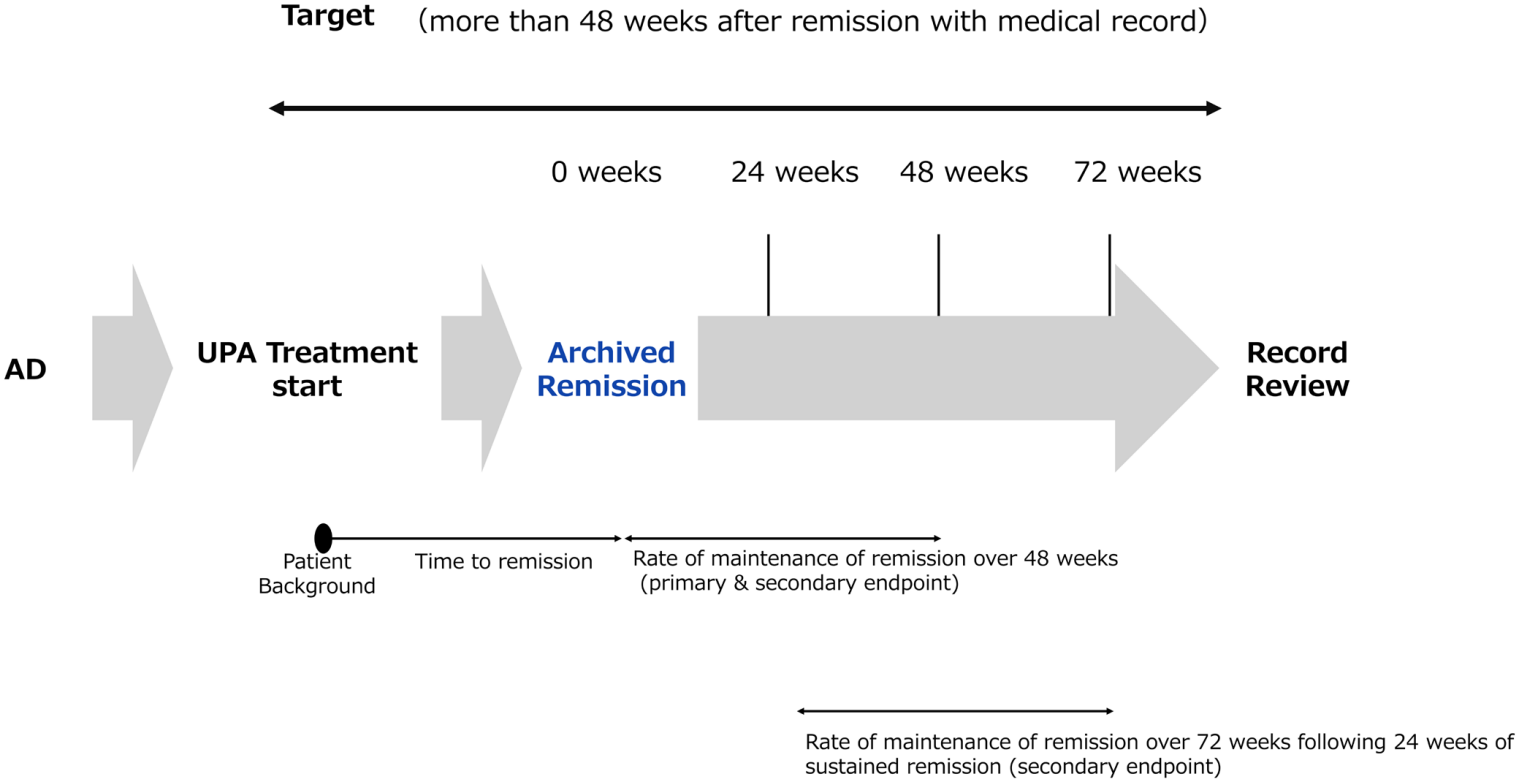
Study design. Retrospective observational study in patients with atopic dermatitis (AD) treated with upadacitinib (UPA) in Japan, evaluating outcomes for at least 48 weeks after achieving remission. Primary and secondary endpoints are indicated.

ROADMAP is a multicenter, retrospective, longitudinal, medical chart review‐based observational study documenting UPA treatment patterns in patients with AD who achieved protocol‐defined remission (*predefined as IGA 0/1 at two consecutive visits* ≥ *4 weeks apart*). It evaluates the maintenance of remission during the ≥ 48‐week period following the initial remission with UPA (Figure 1).

The diagnosis and severity of AD were assessed in routine clinical practice by the treating physicians in accordance with 2024 Japanese guidelines [1]. At each visit, the treating dermatologist performed a routine whole‐body skin examination and assigned IGA on a 0–4 scale according to the standardized scoring instruction [10]. Because this was a retrospective chart‐review study and assessments were performed as part of routine care, no centralized rater‐training was conducted; nevertheless, all sites used the same IGA definitions and the study protocol prespecified outcome definitions to minimize inter‐site variability. Unless otherwise specified, “maintenance of remission” denotes persistence of IGA 0/1 during follow‐up. Relapse was defined as IGA ≥ 2 (i.e., any loss of remission), a conservative threshold; to facilitate cross‐study comparison, exploratory analyses also reported protocol‐defined disease control (maintenance of an IGA ≤ 2) and loss of control (IGA ≥ 3).

During the follow‐up, patients were classified into three dosing‐strategy subgroups: (1) On‐label–continued the Japanese‐approved once daily (QD) dose (15 or 30 mg) [11] throughout follow‐up; (2) Spacing/reduction–extended the dosing interval (e.g., alternate‐day dosing) and/or underwent a dose‐reduction; and (3) Discontinuation–stopped UPA at any point after achieving remission. Patients who initiated other systemic therapy after remission were treated as “switches” and excluded from comparisons between‐groups.

### 2.3 Data Collection and Endpoints

Data were retrospectively collected from medical records by site investigators or designated study staff using paper‐based case report forms or an electronic data capture system. Collected data included demographics, baseline characteristics, treatment history, UPA dosing and modification, clinical responses, use of concurrent topical agents and moisturizers, and adverse events (AEs) over the follow‐up period. The primary endpoint was the maintenance of remission (IGA 0/1) over 48 weeks. Secondary endpoints included the time to relapse, outcomes after prescription changes, and remission status up to 72 weeks following an initial ≥ 24 weeks of sustained remission.

### 2.4 Statistical Analysis

Continuous variables were summarized as mean ± standard deviation (SD). Categorical variables were summarized as frequencies and percentages with 95% confidence intervals (CI). Time‐to‐event outcomes (time to remission and time to relapse) were summarized using the Kaplan–Meier method; fixed‐timepoint estimates (e.g., 48 and 72 weeks) are reported with 95% CI, and group comparisons used the log‐rank test. Exploratory analyses used the Wilcoxon rank‐sum test (non‐parametric) to assess the association between the duration of continuous on‐label treatment prior to modification and subsequent relapse risk, given small subgroup sizes and non‐normal distributions. No imputation of missing values was performed; censored observations were handled per the Kaplan–Meier method. As an exploratory post hoc analysis, patients were categorized into on‐label, spacing/reduction, and discontinuation groups, and outcomes were evaluated accordingly. No adjustment for multiple comparisons was performed; therefore, *p* values should be interpreted descriptively. All statistical analyses were performed using JMP Ver.18 (JMP Statistical Discovery LLC, Cary, NC, USA).

## 3 Results

### 3.1 Backgrounds of the Study Population

A total of 219 patients with AD were included in the study. The mean age ± SD was 36.2 ± 21.3 years; the most common age groups were 18–64 years (56.2%) and 12–17 years (32.4%). Overall, 63.5% had a history of childhood‐onset AD (onset ≤ 15 years) (Table 1).

**TABLE 1:** Baseline characteristics at upadacitinib initiation.

	Number of patients (*n* = 219)	Ratio (%)
Sex: male/female	129/90	58.9/41.1
Age (mean ± SD)	36.2 ± 21.3	
Range		
12–17 years (%)	71	32.4
18–64 years (%)	123	56.2
Over 65 years (%)	25	11.4
Time of AD onset		
< 1 years	25	11.4
1–5 years	74	33.8
6–15 years	40	18.3
Over 16 years	59	26.9
Unknown	21	9.6
IGA score		
IGA 0/1	0	0
IGA 2	11	5.0
IGA 3	152	69.4
IGA 4	56	25.6
Topical anti‐inflammatory agents^a^	195	89.0
Topical corticosteroids	177	80.8
Delgocitinib ointment	52	23.7
Tacrolimus ointment	33	15.1
Difamilast ointment	7	3.2
Moisturizer	159	72.6
Antihistamine	119	54.3

Abbreviations: AD, atopic dermatitis; IGA, Investigator's Global Assessment.

^a^ Including simultaneous use.

At the initiation of UPA treatment, 95.0% had an IGA 3 or 4, with 69.4% classified as IGA 3 (moderate). Most patients (89.0%) were receiving topical anti‐inflammatory agents–most commonly TCS (80.8%), followed by delgocitinib (23.7%) and tacrolimus (15.1%); 72.6% used moisturizers and 54.3% took antihistamines (Table 1). Prior systemic therapy included dupilumab (21.0%), oral corticosteroids (10.0%), cyclosporine (9.6%), and other JAK inhibitors (7.3%). Phototherapy had been used in 6.8% of patients, and 6.8% had a prior AD‐related hospitalization (Table 2).

**TABLE 2:** Prior systemic therapies and other history.

Item	*n* (%)
Prior systemic therapies^a^	
Dupilumab	46 (21.0)
Oral corticosteroids	22 (10.0)
Cyclosporine	21 (9.6)
Oral JAK inhibitor	16 (7.3)
Prior phototherapy	15 (6.8)
Prior hospitalization for AD	15 (6.8)

Abbreviations: AD, atopic dermatitis; JAK, Janus kinase.

^a^ Simultaneous use allowed.

Lifestyle data, including smoking and alcohol consumption habits, were only available for 141 patients aged ≥ 20 years (Table S1), but no notable differences in lifestyle factors were observed between sex groups.

### 3.2 Initial Achievement of Remission With UPA Treatment

**FIGURE 2:**
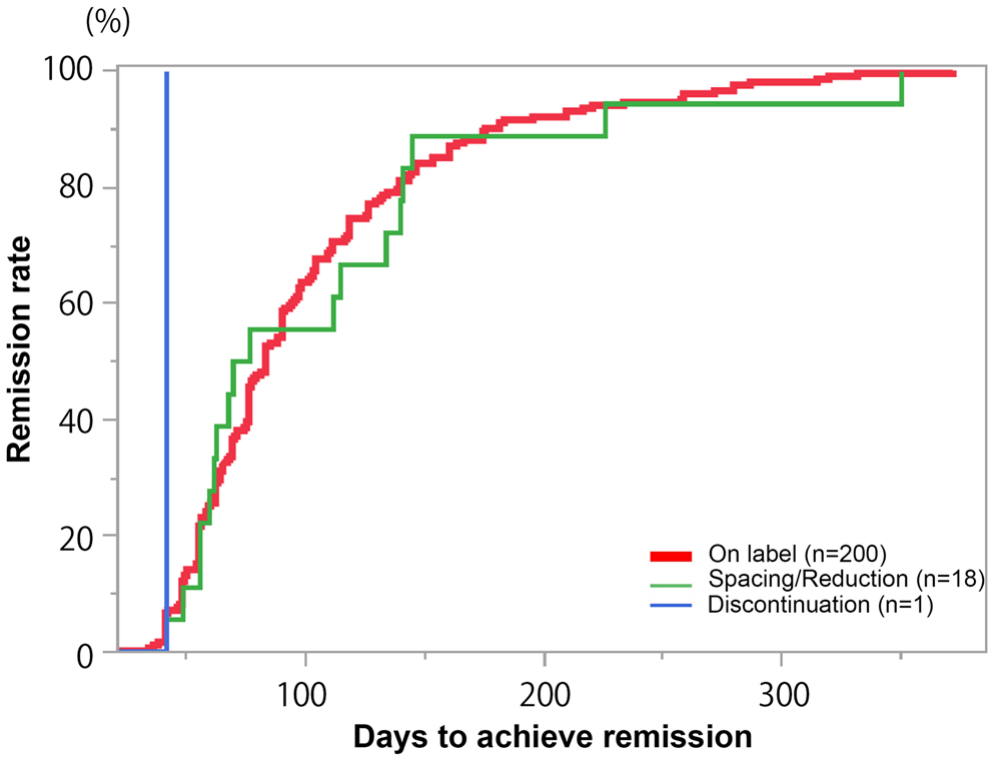
Time to remission after upadacitinib initiation. Kaplan–Meier curves showing time to remission by prescription pattern: On‐label (red), spacing/reduction (green), and discontinuation (blue). Remission was defined as an Investigator's Global Assessment (IGA) score of 0 or 1 at two consecutive visits ≥ 4 weeks apart. Log‐rank test: *p* = 0.72 (on‐label vs. spacing/reduction).

Of the 219 patients who achieved remission with UPA, 91.3% (*n* = 200) were prescribed the Japanese‐approved once‐daily (QD) dose (on‐label subgroup) (Figure 2). Within this on‐label subgroup, 140 patients remained on 15 mg QD, 42 on 30 mg QD, 6 escalated from 15 → 30 mg QD, 10 de‐escalated from 30 → 15 mg QD, and 2 alternated 15 and 30 mg QD on alternate days. A further 8.2% (*n* = 18) achieved remission under a spacing/reduction regimen (dose‐reduction or extended dosing interval, e.g., alternate‐day dosing). One patient (0.5%) achieved remission despite complete discontinuation between the remission‐defining visits.

In time‐to‐event analyses, the on‐label subgroup achieved remission after 103.5 ± 4.4 days (25th/50th/75th percentiles: 60.5/84.0/126.5 days), indicating that most patients reached remission within approximately 4 months of initiation. The spacing/reduction subgroup had a mean time to remission of 109.2 ± 18.1 days (25th/50th/75th percentiles: 60.0/73.5/140.0 days), with no significant difference versus on‐label subgroup (log‐rank *p* = 0.72).

### 3.3 Maintenance of Remission Over 48 Weeks Following Initial Remission (Among Initial UPA Responders)

**FIGURE 3:**
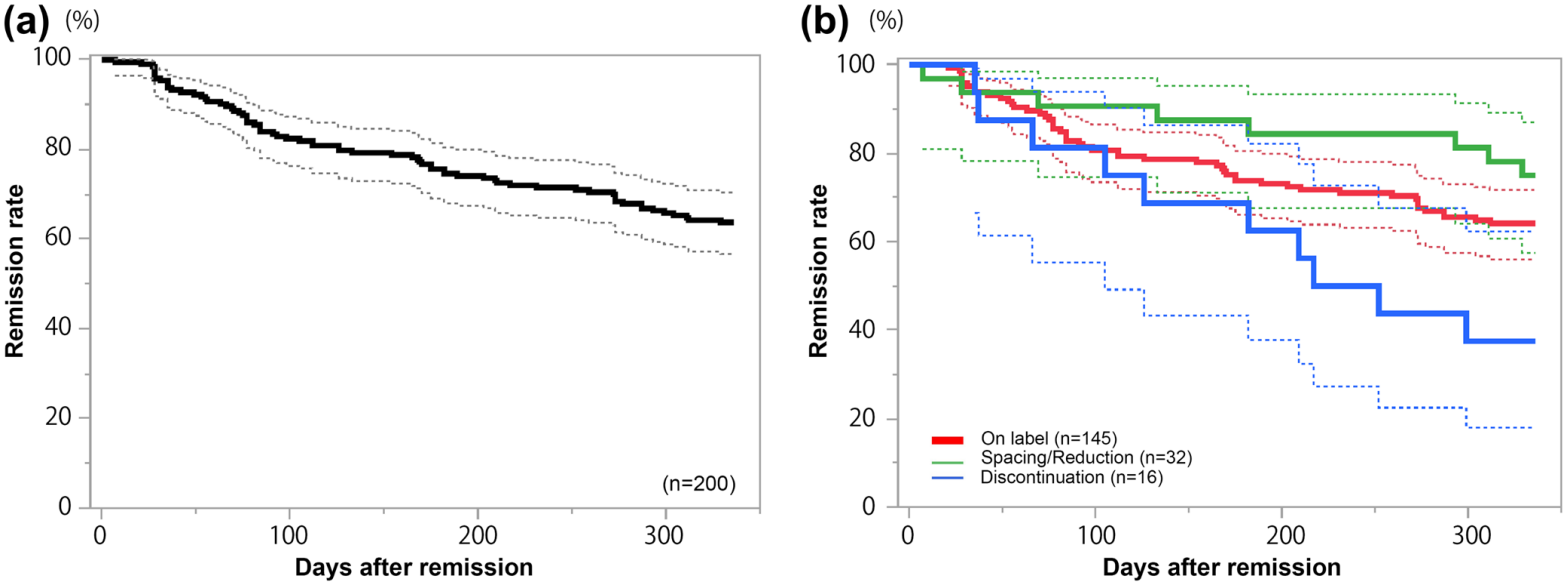
Proportion maintaining remission for 48 weeks after remission. Kaplan–Meier curves of remission maintenance (IGA 0/1). (a) All patients (*n* = 200). (b) By prescription pattern (excluding 7 who switched to other systemic therapy): On‐label (red), spacing/reduction (green), discontinuation (blue). Dotted lines indicate 95% confidence intervals (CI). *p* values by log‐rank test.

Among the 200 patients who achieved initial remission with on‐label UPA and were eligible for follow‐up, 127 (63.5%, 95% CI 56.6–69.9) maintained remission (IGA 0/1) throughout the 48 weeks (Figure 3a). When stratified by dosing strategy (*n* = 193; 7 switched to another systemic therapy after remission and were excluded), remission at 48 weeks was maintained in 93/145 (64.1%, 95% CI 55.8–71.9) in the on‐label group, 24/32 (75.0%, 95% CI 56.6–88.5) in the spacing/reduction group, and 6/16 (37.5%, 95% CI 15.2–64.6) in the discontinuation group (Figure 3b). Between‐group comparisons are descriptive only and may be affected by confounding by indication.

**FIGURE 4:**
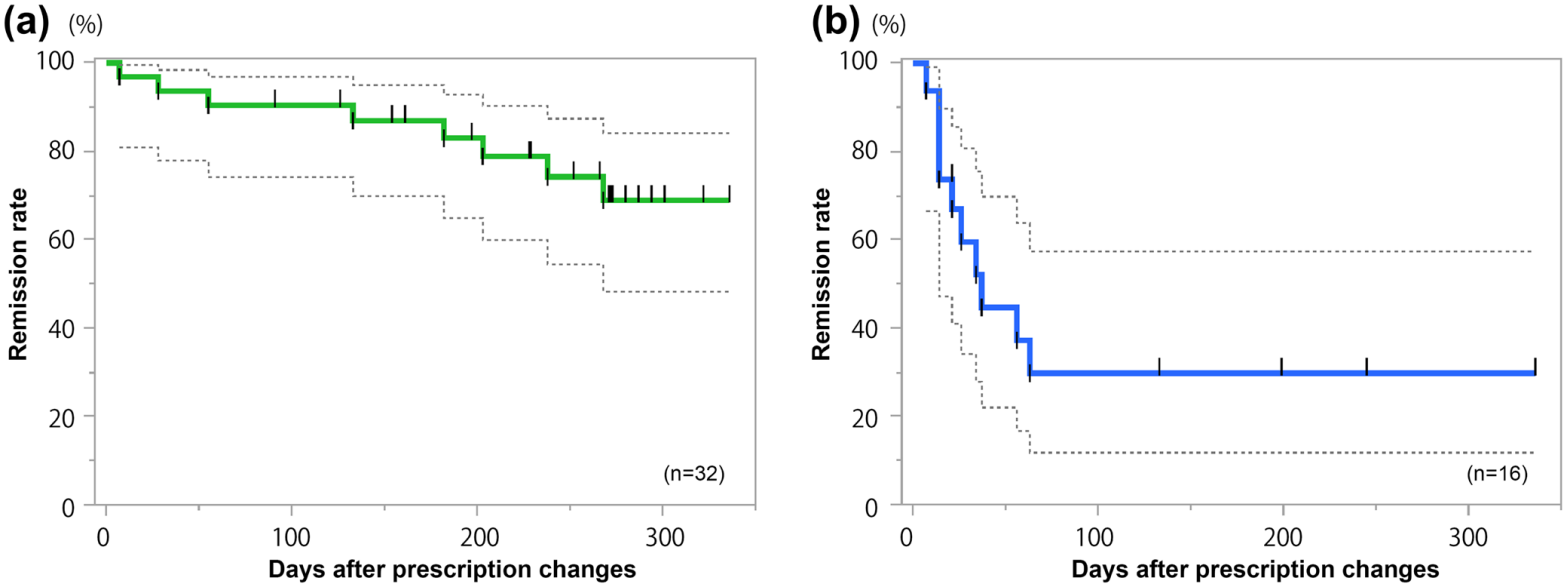
Remission after prescription changes. Kaplan–Meier curves of remission maintenance after treatment modification. (a) Spacing/reduction (green). (b) Discontinuation (blue). Dotted lines indicate 95% CI; vertical ticks indicate censored cases.

**FIGURE 5:**
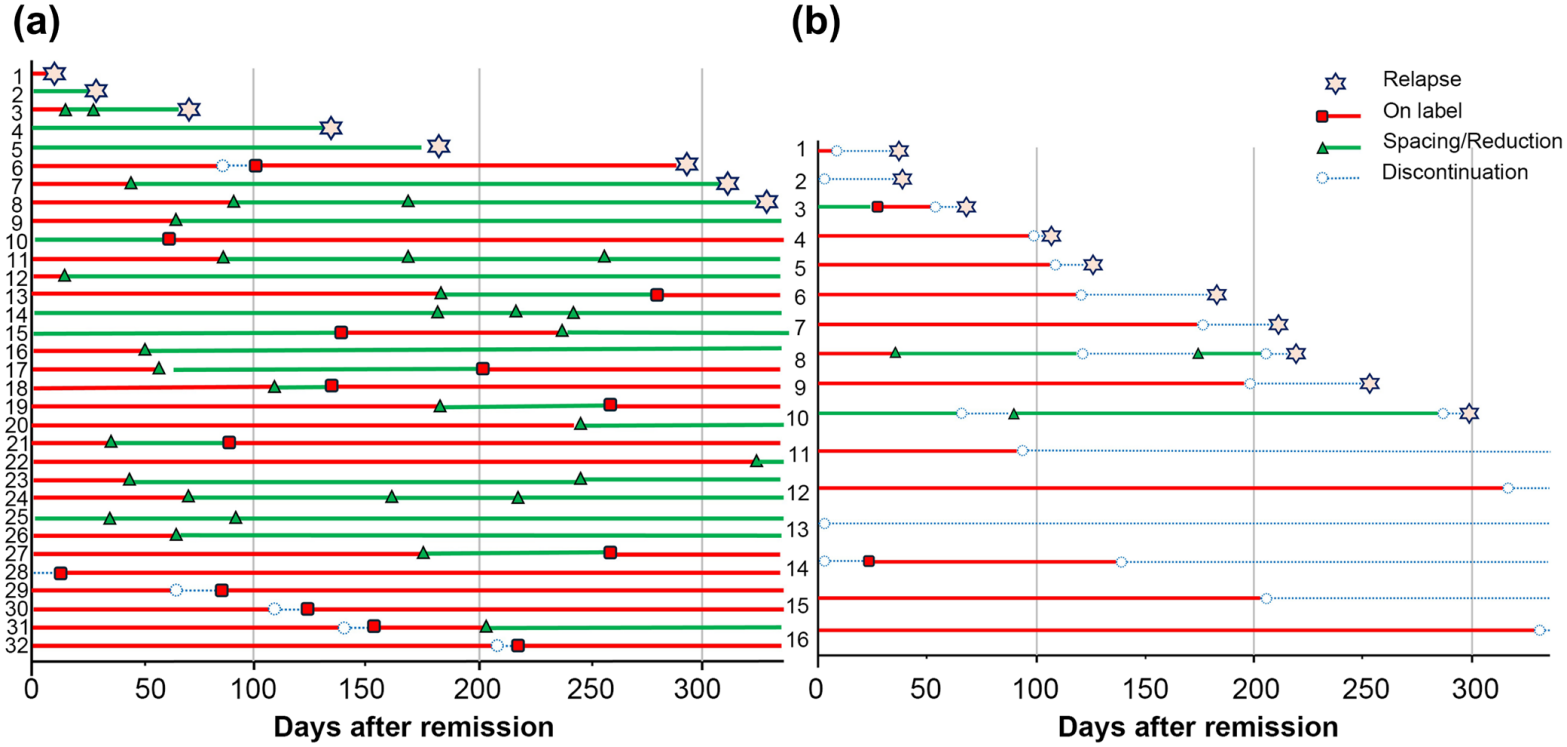
Case‐by‐case treatment trajectories (swimmer plots). Individual treatment courses following remission. (a) Spacing/reduction group (*n* = 32). (b) Discontinuation group (*n* = 16). Relapse events (IGA ≥ 2), when observed, are indicated on the plots.

In an exploratory analysis 181 of 200 patients (90.5%, 95% CI 85.6–93.9) maintained “disease control” (IGA ≤ 2) over 48 weeks (Figure S1). Remission after treatment modification (starting from the day of change) was further evaluated; the remission rate was 68.9% in the spacing/reduction group (Figure 4a) and 29.8% in the discontinuation group (Figure 4b). The most frequent interval modification was alternate‐day dosing; other patterns included twice‐weekly dosing and a “4 days on/1 day off” regimen (Figure 5a,b).

**FIGURE 6:**
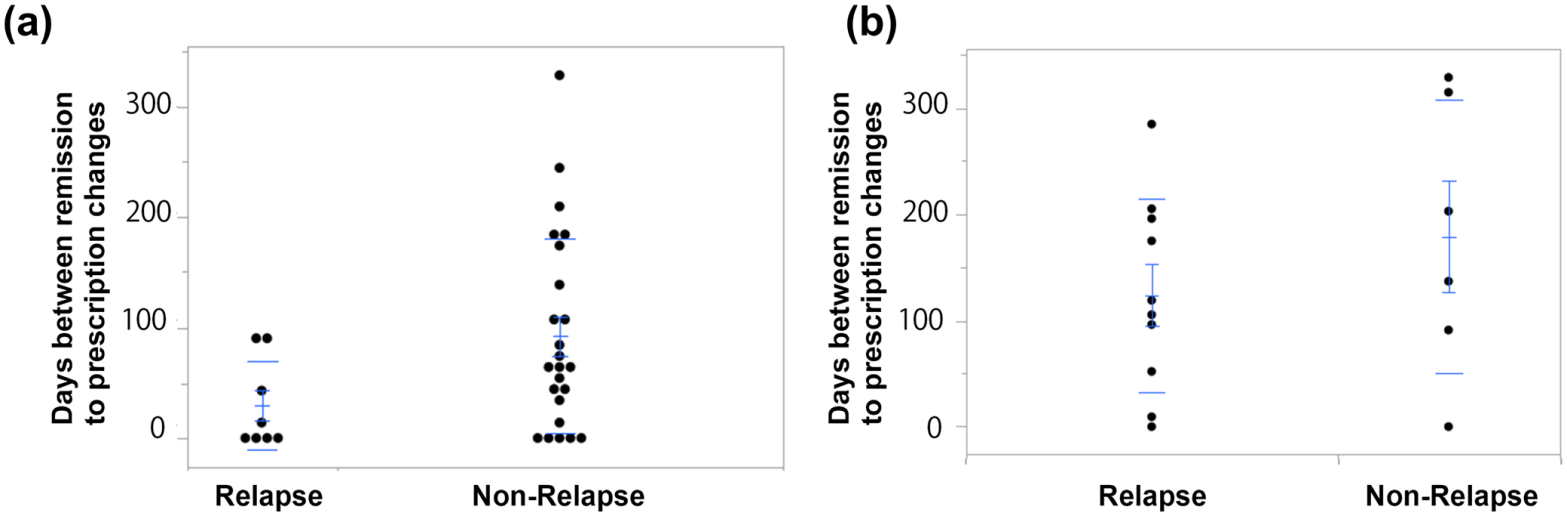
Impact of pre‐change on‐label duration on relapse. Dot plots showing days of continuous on‐label UPA before treatment modification. (a) Spacing/reduction group (relapse, *n* = 8; non‐relapse, *n* = 24). (b) Discontinuation group (relapse, *n* = 10; non‐relapse, *n* = 6). Bars indicate the mean ± standard deviation (SD; long blue bars) and the mean ± standard error (SE; short blue bars).

Among patients who underwent dose modification or discontinuation, those without loss of remission tended to have a longer on‐label period prior to the change than those with loss of remission. In the spacing/reduction subgroup, the on‐label duration before change was 92.5 ± 88.3 days in non‐relapsers versus 29.8 ± 40.3 days in relapsers (*p* = 0.0645, Wilcoxon; Figure 6a). In the discontinuation subgroup, the corresponding durations were 179.2 ± 128.9 versus 124.2 ± 91.2 days (*p* = 0.4153; Figure 6b).

Baseline characteristics by relapse status are shown in Table 3; no material differences were observed in age, baseline IGA, time to remission, or age at disease onset.

**TABLE 3:** Patient characteristics by relapse status in the spacing/reduction and discontinuation groups.

Variable	Spacing/Reduction		Discontinuation	
	Relapse (*n* = 8)	Non‐relapse (*n* = 24)	Relapse (*n* = 10)	Non‐relapse (*n* = 6)
Age, years (mean ± SD)	46.4 ± 24.6	45.9 ± 22.6	40.1 ± 21.2	28.2 ± 14.3
IGA score (mean ± SD)	3.4 ± 0.5	3.3 ± 0.6	3.1 ± 0.6	3.2 ± 0.4
Days to remission (mean ± SD)	84.9 ± 38.0	70.8 ± 25.6	90.5 ± 47.0	91.3 ± 31.4
Other allergies, *n* (%)	3 (37.5%)	11 (45.8%)	4 (40.0%)	3 (50.0%)
Medical history, *n* (%)	0 (0.0%)	7 (29.2%)	2 (20.0%)	2 (33.3%)
Comorbidities, *n* (%)	5 (62.5%)	10 (41.7%)	3 (30.0%)	1 (16.7%)
Age of AD onset, *n* (%)				
< 1 year	3 (37.5%)	1 (4.2%)	1 (10.0%)	0 (0.0%)
1–5 years	1 (12.5%)	7 (29.2%)	4 (40.0%)	3 (50.0%)
6–15 years	1 (12.5%)	5 (20.8%)	3 (30.0%)	1 (16.7%)
≥ 16 years	2 (25.0%)	11 (45.8%)	1 (10.0%)	1 (16.7%)
Unknown	1 (12.5%)	0 (0.0%)	1 (10.0%)	1 (16.7%)
Concomitant topical anti‐inflammatory drugs, *n* (%)	7 (87.5%)	22 (91.7%)	9 (90.0%)	5 (83.3%)

*Note:* Values are shown as mean ± SD or *n* (%) as appropriate.

Abbreviations: AD, atopic dermatitis; IGA, Investigator's Global Assessment.

### 3.4 Remission Rate at 72 Weeks Following 24 Weeks of Sustained Remission

A total of 123 patients receiving on‐label treatment maintained a remission response (IGA 0/1) for 24 consecutive weeks. Three patients switched to another systemic therapy after maintaining remission for 24 consecutive weeks and were excluded from between‐group comparisons. Among the remaining patients, the distribution by treatment course subgroup up to week 72 was as follows: 87.0% (*n* = 107) remained on on‐label treatment, 6.5% (*n* = 8) were in the spacing/reduction group, and 4.1% (*n* = 5) were in the discontinuation group.

**FIGURE 7:**
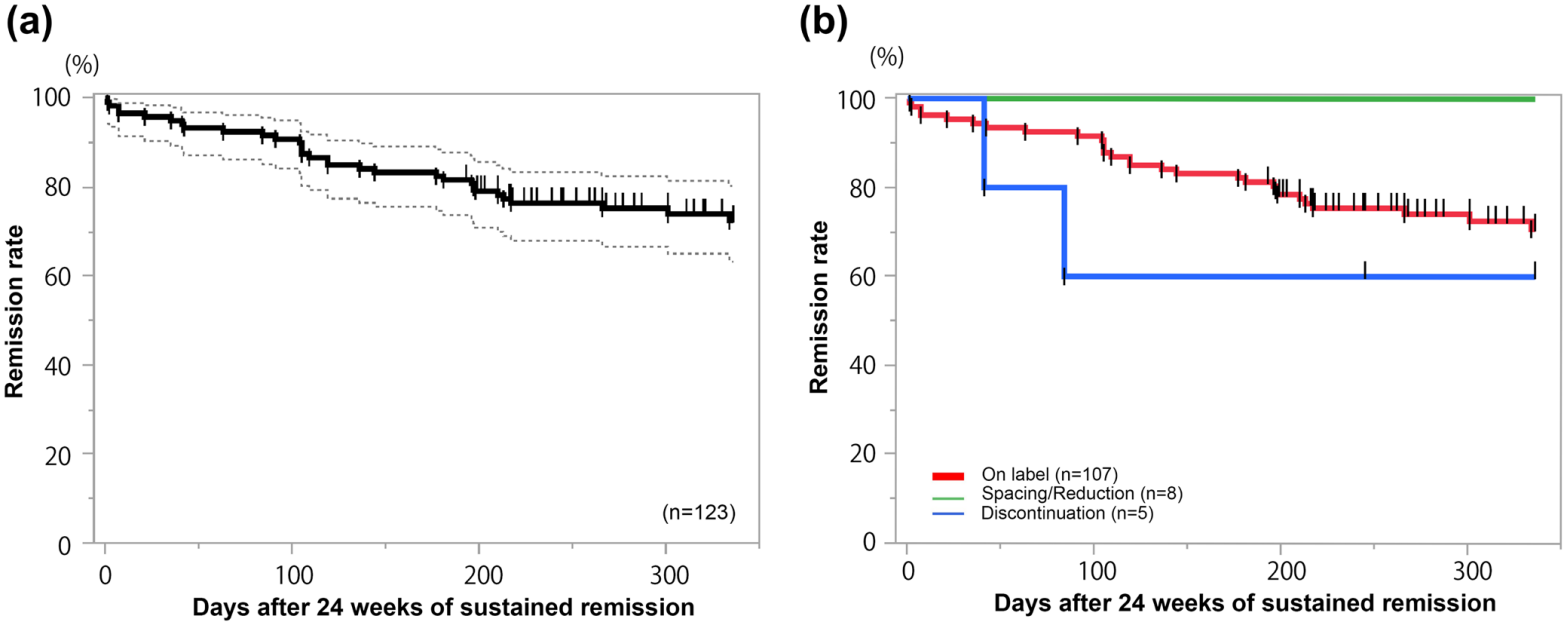
Remission at 72 weeks following 24 weeks of sustained remission. Kaplan–Meier curves of remission maintenance (IGA 0/1). (a) All patients (*n* = 123). (b) By prescription pattern (excluding 3 who switched to other therapy): On‐label (red), spacing/reduction (green), discontinuation (blue). Dotted lines indicate 95% CI; vertical ticks indicate censored cases.

At week 72 (i.e., 48 weeks after the 24‐week sustained‐remission mark), the overall remission maintenance rate was 72.3% (95% CI 63.2–79.9) (Figure 7a). When stratified by treatment course subgroup, remission was maintained in 70.7% (95% CI 60.7–79.1; *n* = 107) of the on‐label subgroup, 100% (95% CI 63.1–100.0; *n* = 8) of the spacing/reduction subgroup, and 60.0% (95% CI 20.0–90.0; *n* = 5) of the discontinuation subgroup (Figure 7b); estimates for spacing/reduction and discontinuation were imprecise due to the limited sample size.

### 3.5 Safety Evaluation

AEs were reported throughout the follow‐up period (Table 4). The most frequently reported AEs were acne (*n* = 31), herpes zoster (*n* = 12), herpes simplex (*n* = 11), and folliculitis (*n* = 8). Most events were mild and were managed with symptomatic treatment. One herpes zoster case was serious and required a temporary treatment interruption; the patient resumed UPA after full recovery without recurrence. No cases of malignancy, thrombosis, or laboratory abnormalities requiring intervention were reported. The incidence of AEs did not differ meaningfully among the on‐label, spacing/reduction, and discontinuation groups.

**TABLE 4:** Adverse events (121 events in 76 patients).

Adverse event	Patients, *n* = 219 (%)	Events, *n*	Severity, *n*	Treatment discontinuation due to AE, *n*
Acne	31 (14.2)	36	Mild: 30, Moderate: 6	1
Herpes zoster^a^	12 (5.5)	14	Mild: 5, Moderate: 8, Severe: 1	1
Herpes simplex	11 (5.0)	22	Mild: 20, Moderate: 2	
Folliculitis	8 (3.7)	11	Mild: 10, Moderate: 1	
Hepatic function abnormal	5 (2.3)	5	Mild: 4, Moderate: 1	2
Diarrhea	2 (0.9)	2	Mild	
Headache	2 (0.9)	2	Mild: 1, Moderate: 1	
Abdominal pain	1 (0.5)	2	Mild: 2	1
Eyelid oedema	1 (0.5)	2	Moderate: 2	1
COVID‐19	1 (0.5)	1	Mild	
Furuncle	1 (0.5)	1	Mild	
Molluscum contagiosum	1 (0.5)	1	Mild	
Bacterial skin infection	1 (0.5)	1	Mild	
Tonsillitis	1 (0.5)	1	Moderate	
Corneal infection	1 (0.5)	1	Moderate	
Paronychia	1 (0.5)	1	Mild	
Eczema	1 (0.5)	1	Moderate	
Toxic skin eruption	1 (0.5)	1	Mild	
Erythema	1 (0.5)	1	Moderate	
MAFLD (fatty liver disease)	1 (0.5)	1	Mild	1
Eyelid erythema	1 (0.5)	1	Mild	
Cough	1 (0.5)	1	Mild	
Oropharyngeal pain	1 (0.5)	1	Mild	
Renal impairment	1 (0.5)	1	Mild	
CPK increased	4 (1.8)	4	Mild: 2, Moderate: 2	2
WBC decreased	1 (0.5)	2	Mild: 2	
GGT increased	1 (0.5)	1	Mild	1
ALT increased	1 (0.5)	1	Mild	
Platelets increased	1 (0.5)	1	Moderate	
Creatinine increased	1 (0.5)	1	Mild	

Abbreviations: ALT, alanine aminotransferase; CPK, creatine phosphokinase; GGT, gamma‐glutamyl transferase; MAFLD, metabolic dysfunction‐associated fatty liver disease; WBC, white blood cell.

^a^ One serious case (herpes zoster).

## 4 Discussion

This multicenter, retrospective real‐world study characterizes long‐term outcomes after protocol‐defined remission (IGA 0/1 at two consecutive visits) among Japanese patients with AD treated with UPA. Because the analysis was restricted to responders who achieved remission, the reported maintenance rates may overestimate remission durability in the overall UPA‐treated population. Remission was maintained through 48 weeks in 63.5% overall; by post‐remission strategy, maintenance at 48 weeks was 64.1% with continued on‐label dosing, 75.0% with dose‐interval extension or dose‐reduction, and 37.5% with complete discontinuation. These between‐group differences are descriptive only and should be interpreted cautiously due to potential selection bias and confounding by indication. The spacing/reduction subgroup was smaller, and decisions to space/reduce doses in routine practice are heterogeneous–often made after stable disease control but sometimes prompted by tolerability/safety concerns or patient preference. Among patients with ≥ 24 weeks of sustained remission, 72‐week maintenance was 70.7% on‐label (*n* = 107), 100% with spacing/reduction (*n* = 8; 95% CI 63.1–100.0), and 60.0% with discontinuation (*n* = 5), although estimates were imprecise due to the limited sample size. These findings provide practical insight for maintenance and modification under routine care.

Importantly, these observations align with current guidance in Japan, which recommends reassessing the need for continued therapy after approximately 24 weeks of sustained remission, consistent with current Japanese guidelines [1, 9]. When de‐escalation is contemplated at that reassessment, our data suggest that dose‐interval extension or dose‐reduction may be a more prudent intermediate step than abrupt cessation, given the higher risk of loss of remission observed with immediate discontinuation at both 48 and 72 weeks. Patients who modified treatment soon after remission were more likely to relapse, supporting the practicality of consolidating remission on on‐label dosing before attempting spacing/reduction.

Time to initial remission also informs clinical expectations: the median was 84 days (~12 weeks), with an interquartile range indicating that many—but not all—patients achieve remission within ~3–4 months. This distribution argues for allowing an adequate therapeutic window before concluding non‐response, while concurrently planning maintenance and, where appropriate, potential de‐escalation once remission stabilizes.

Our interpretation is consistent with the broader evidence base. PhaseIII studies, including the AD Up trial evaluating UPA in combination with TCS, have confirmed its efficacy and safety across different treatment settings [12]. Moreover, a recent phase IIIb/IV randomized treat‐to‐target trial (Flex Up) demonstrated that protocolized dose escalation from UPA 15 to 30 mg in patients who had not achieved EASI 90 at week 12, and dose‐reduction from 30 to 15 mg in those who had achieved EASI 90, enabled many patients to newly reach or maintain EASI 90 through week 24 without new safety signals [13]. This supports the concept that structured dose modification, rather than abrupt discontinuation, can sustain disease control in appropriately selected responders, in line with the pattern observed in our cohort.

Furthermore, randomized withdrawal data for other oral JAK1 inhibitors indicate that discontinuation leads to earlier and more frequent flares than continued therapy or dose‐reduction [14]. Because flare/relapse definitions differ (e.g., IGA ≥ 3 in some trials), whereas the present study defined relapse as IGA ≥ 2 (any loss of remission), direct numerical comparisons should be made cautiously; this conservative definition may yield lower apparent remission‐maintenance rates. To aid interpretation and cross‐study comparison, we also reported IGA ≤ 2 (disease control) and IGA ≥ 3 (loss of control) as exploratory outcomes [14, 15].

Safety findings were consistent with prior trials in Japanese and global populations: acne, herpes zoster, and herpes simplex were most frequent and predominantly mild; one serious herpes zoster event required temporary interruption with successful resumption [4, 6, 8, 15]. However, safety outcomes should be interpreted in light of the responder‐enriched study population and the observational follow‐up duration; early discontinuations and rare or delayed‐onset adverse events may be underrepresented. Taken together with long‐term post‐marketing surveillance, these results support a favorable benefit–risk profile of UPA in real‐world practice while underscoring the need for continued long‐term follow‐up to monitor rare or delayed‐onset events [16].

From a therapeutic‐strategy standpoint, these data support a structured, individualized plan after remission: (i) reassess the need for continued therapy after sustained remission, as recommended by national guidelines [1, 9]; (ii) if discontinuation is being considered, prefer dose‐interval extension or dose‐reduction as an intermediate step rather than abrupt cessation, with shared decision‐making and a predefined rescue plan; and (iii) acknowledge that spacing schedules varied in practice (alternate‐day, twice‐weekly, “4‐days‐on/1‐day‐off,” etc.), and our study was not powered to determine an optimal regimen. Regarding rescue strategies, previous real‐world data indicate that reintroduction of UPA after relapse is generally effective and safe, which supports the feasibility of discontinuation trials in carefully selected patients. Within these limits, the overall pattern that tapering/spacing before stopping performs better than immediate stopping is clinically useful and congruent with the guideline‐based approach to reassessment and individualized long‐term management [1, 6].

## 5 Limitations

This retrospective chart‐review design is subject to selection bias, missing data, and ascertainment/reporting variability. Medication records may not fully capture adherence or the granularity of spacing patterns. Treatments for comorbidities (including potentially immunosuppressive medications) were not systematically collected, and therefore their potential influence on relapse risk could not be evaluated. By design, we evaluated initial UPA responders, limiting generalizability to all treated patients and likely inflating estimates of remission durability and tolerability. IGA assessments were performed by multiple treating physicians in routine care without centralized rater‐training, which may introduce interobserver variability. Subgroup sizes–especially for spacing/reduction and discontinuation–were small, and comparisons between groups are descriptive rather than inferential and remain vulnerable to confounding by indication; confidence intervals were wide for small subgroups. Finally, outcome thresholds differ across studies (relapse defined as IGA ≥ 2 here vs. IGA ≥ 3 in some randomized withdrawal trials), constraining cross‐study comparisons [14, 15]. Follow‐up duration may also have been insufficient to detect rare or long‐latency adverse events.

## 6 Conclusions

In routine Japanese practice, most patients treated with UPA who achieved protocol‐defined remission maintained IGA 0/1 over the subsequent 48 weeks. In this responder‐enriched cohort, continued on‐label dosing and cautious dose spacing/reduction were associated with durable remission, whereas discontinuation was associated with a higher observed risk of loss of remission; however, due to the retrospective design, responder‐only selection, small subgroup sizes, and confounding by indication, these findings should be considered descriptive and hypothesis‐generating. Prospective studies are needed to validate optimal long‐term maintenance and de‐escalation strategies and to identify predictors of successful tapering, spacing, or discontinuation.

Prospective studies are warranted to validate these observations and to further define optimal long‐term AD treatment strategies, including standardized criteria for remission and relapse and longer‐term safety follow‐up.

Figure S1 shows Kaplan–Meier estimates of disease control (IGA ≤ 2) over 48 weeks after remission, providing complementary information to the primary remission‐maintenance analysis based on IGA 0/1.

## Funding

This work was supported by AbbVie.

## Ethics Statement

This study was conducted in accordance with the Ethical Guidelines for Medical and Health Research Involving Human Subjects in Japan and the principles of the Declaration of Helsinki. The study protocol was reviewed and approved by the Institutional Review Board of the NPO Health Institute Research of Skin (approval date: 26 March 2024; IRB number: 19000025).

## Consent

Informed consent was obtained in the form of an opt‐out on the website of each participating institution. Patients who declined to participate were excluded from the study. The study is registered with the University Hospital Medical Information Network (UMIN000054106).

## Conflicts of Interest

Y.T. has received consulting fees or speaker honoraria from KAKEN pharmaceutical, Eisai, Maruho, Sato Pharmaceutical, Janssen Pharmaceutical, Sun Pharma, Sanofi, Eli Lilly, AbbVie, UCB Japan, Pfizer, LEO pharma, Otsuka Pharmaceutical, Torii Pharmaceutical and TAIHO Pharmaceutical. Y.T. is an Editorial Board member of The Journal of Dermatology and a co‐author of this article. A.I. has received advisory board honoraria, consulting fees or speaker honoraria from AbbVie, Eli Lilly, Japan Tobacco, Maruho, Novartis, Sanofi, LEO pharma, and TORII Pharmaceutical; he has also received research grants from AbbVie, Eli Lilly, Japan Tobacco, Novartis, Otsuka Pharmaceutical, Amgen, and Sanofi. H.S. has received speaker's fees from AbbVie, Sanofi, Bristol‐Myers Squibb, Eli Lilly, Maruho, Nippon Boehringer Ingelheim, Taiho Pharmaceutical, Otsuka Pharmaceutical, Pfizer Japan, TORII Pharmaceutical, UCB Japan, Amgen, and Novartis; research grants (clinical trials) from AbbVie and LEO Pharma; scholarships from Sun Pharma Japan, Maruho, Taiho Pharmaceutical, Tokiwa Pharmaceutical, Eisai, and TORII Pharmaceutical; and is an Editorial Board member of The Journal of Dermatology and a co‐author of this article. To minimize bias, Y.T. and H.S. were excluded from all editorial decision‐making related to the acceptance of this article for publication. T.I. is an employee of AbbVie Inc.

## Supporting information


**Figure S1:** Disease control (IGA ≤ 2) over 48 weeks after remission. Kaplan–Meier curves showing the proportion of patients maintaining IGA ≤ 2. Loss of control was defined as IGA ≥ 3. Dotted lines indicate 95% CI.

**Table S1:** Lifestyle factors in adults (≥ 20 years) by sex.

## Data Availability

The data underlying this article cannot be shared publicly to protect the privacy of individuals who participated in the study. The data will be shared upon reasonable request from the corresponding authors.
