# Supplementary material for: Real‐World Maintenance of Remission in Atopic Dermatitis Patients With Upadacitinib: A Multicenter Retrospective Study in Japan (ROADMAP Study)
Source: J Dermatol. 2026 Jun 4;53(7):980–9. doi: 10.1111/1346-8138.70299 (PMC13341070; doi:10.1111/1346-8138.70299)
Supplement: Supplementary file 2 — Table S1: Lifestyle factors in adults (≥ 20 years) by sex. [file JDE-53-980-s001.docx]

Supplementary

**Table S1. Lifestyle factors in adults (≥20 years) by sex**

|  | **Total n=141** | **Male n=81** | **Female n=60** |
| --- | --- | --- | --- |
| **Smoking** |  |  |  |
| Current | 11 (7.8) | 8 (9.9) | 3 (5.0) |
| Former | 10 (7.1) | 10 (12.3) | 0 (0.0) |
| Never | 51 (36.2) | 23 (28.4) | 28 (46.7) |
| Unknown | 69 (48.9) | 40 (49.4) | 29 (48.3) |
| **Alcohol** |  |  |  |
| Present | 27 (19.1) | 21 (25.9) | 6 (10.0) |
| Absent | 43 (30.5) | 18 (22.2) | 25 (41.7) |
| Unknown | 71 (50.4) | 42 (51.9) | 29 (48.3) |
